# Supplementary material for: Sustained-input switches for transcription factors and microRNAs are central building blocks of eukaryotic gene circuits
Source: Genome Biol. 2013 Aug 23;14(8):R85. doi: 10.1186/gb-2013-14-8-r85 (PMC4054853; doi:10.1186/gb-2013-14-8-r85)
Supplement: Additional file 5 — HTML Browsable Motif Output. Zipped folder containing all WaRSwap and FANMOD motif output, viewable in a web browser. [file gb-2013-14-8-r85-S5.ZIP › HTML_browsable_motif_output/WaRSwap_all_tair9/sigs_WSWRSLDraw-2000_all.pvals.heatmaps.html/motif_id_174_011101101_tftype_all_upstream_-2000_0.html]

```
BG_MODEL = Rnew2
MOTIF_ID = 174_011101101
TF_TYPE = all
UPSTREAM = -2000_0


PVals
FN_0.2	FN_0.4	FN_0.6	FN_0.8
dg_60.genes	0.33	0.008	0.14	0.072
dg_70.genes	0.265	0.006	0.121	0.058
dg_80.genes	0.726	0.122	0.46	0.046

ZScores
FN_0.2	FN_0.4	FN_0.6	FN_0.8
dg_60.genes	0.401	2.883	0.877	-0.249
dg_70.genes	0.612	3.147	0.986	-0.226
dg_80.genes	-0.674	1.128	-0.352	-0.201

StDevs
FN_0.2	FN_0.4	FN_0.6	FN_0.8
dg_60.genes	292.469	59.032	5.881	0.356
dg_70.genes	285.01	56.639	5.652	0.301
dg_80.genes	230.015	47.506	4.657	0.271
```
